# Heavy metals pollution and ecological risk assessment around artisanal gold mines in Zamfara, Nigeria

**DOI:** 10.5620/eaht.2024016

**Published:** 2024-06-07

**Authors:** Bate Garba Barde, Adeniyi Olanrewaju Adeleye, Amoo Afeez Oladeji, Yunana Bitrus Duhu

**Affiliations:** 1 Department of Environmental Sciences, Federal University Dutse, Jigawa State, Nigeria; 2 Department of Biological Sciences, University of Maiduguri, Borno State, Nigeria

**Keywords:** Heavy metals, WHO limits, Ecological risk index, Source apportionment

## Abstract

Heavy metals pollution and potential ecological risk index were assessed in artisanal gold mining areas of Zamfara state, Nigeria. Soil samples were collected from three mines namely Kwali (05°45.49'E–11°59.66'N), Duke (06°19.56'E–12°21.45'N) and Maraba (06°22.43'E–12°20.26'N) while a non–mining area; Kadauri (06°08.71'E–12°13.56'N) was also chosen as a control. Samples were analysed using atomic absorption spectrophotometer and the results obtained showed that; the most abundant metal was Pb (148.59 mg/kg) in Kwali and the least was Ni (1.25 mg/kg) in Kadauri while the metals generally occurred in the order; Pb > Fe > Au > Al > Zn followed by other metals in an unspecified manner. All metal concentrations differed significantly (P<0.05) across sampling stations except Mn and Zn and they were all above the WHO limit which portrays a health risk. A strong positive correlation was found between metal pairs with r ≥ 0.5 (*p<0.05) in about 70% of them indicating commonality of source. Pb, Cd, Al and Au had contamination factors > 1in all sampling locations indicating increment in their concentrations above the pre–anthropogenic activities reference levels. Geoaccumulation index showed concentrations above background values of Pb, Cd, Al and Au while potential ecological risk index was highest (782.79) in Kwali and lowest (142.15) in Kadauri. Two principal components accounted for about 99.64% of the total variation in metals concentration with PC1 (95.21%) and PC2 (4.43%). This study showed the influence of artisanal gold mining on heavy metals concentration and suggested regulation of these practices.

## Introduction

Gold is commonly used in the fields of electronics, medicine, decoration, food and space technology as well as some personal care and industrial applications. The high demand and shortage in supply of which leads to its intensified exploration and mining at different levels all over the world [1, 2]. There are several techniques and processes by which gold may be extracted from the earth. These include some primitive methods in which local tools such as digger, pickaxe, shovel and hammer are used and the modern methods in which large-scale gold mining is done using sophisticated machines designed for that purpose [3, 4]. There are approximately 20 million artisanal miners in over 70 countries all over the world who are directly involved in gold mining due to its increasing value, producing about 380 to 450 tonnes per annum [5, 6].

The term heavy metal is often referred to as a group of metals and metalloids that have been associated with contamination and possible toxicity or ecotoxicity [7]. Various factors have been used to define and identify heavy metals, these include; density or specific gravity, atomic weight, atomic number and other chemical properties [8]. They are metals that are persistent in all parts of the environment, generally have densities above 5g/cm3 and cannot be easily degraded or destroyed [9]. Some of these heavy metals such as iron, cobalt, zinc, manganese, and molybdenum are needed for proper functioning of body systems and are required in small amounts, the excess of which can cause harm. Non-essential heavy metals of interest include cadmium, mercury, arsenic and lead, they are part of the ten chemicals that constitute major public health concerns according to the World Health Organization [10, 11]. Unlike organic contaminants which are oxidized to carbon (IV) oxide by microbial action, most heavy metals are not biodegradable and may be toxic to microbes [12]. Their total concentration in soil persists for a long time after their introduction [13], changes in their chemical forms (speciation) and bioavailability are, however possible [14,15]. Organic contaminants’ biodegradation suffers a setback if heavy metals are present in the environment [16]. Gold mining and processing entail a rigorous step by step process where the ground surface is dug to excavate the ore which is crushed and washed with water and/or other chemicals such as mercury and cyanide to concentrate the gold through sluicing, panning, floating and leaching [17]. These activities use local tools or heavy equipments that use gasoline or petrol, as well as explosives such as dynamite and leave behind a lot of by–products ranging from the heap of excavated soil, remains of the ore, wastewater, chemical residues and a host of other wastes which are collectively called tailings [18, 19] and have a great influence on heavy metals concentration in the surrounding soil.

The potential ecological risk index method was advanced by the Swedish scholar, Lars Hakanson and is based on the characteristics of heavy metals and their environmental behavior [20]. It is an approach to evaluate heavy metal contamination from the perspective of sedimentology which not only considers the heavy metal levels in the soil, but also associates the ecological and environmental effects with toxicology and evaluates the pollution level using the comparable and equivalent property index grading method [21, 22]. The pollution coefficients of individual metals, their toxicity response coefficients and the individual metals’ potential ecological risk coefficients are the factors that made up the potential ecological risk index [23]. This study was conducted to assess the pollution levels and potential ecological risk index of heavy metals in soil from artisanal gold mining areas of Zamfara state, Nigeria and carry out a source apportionment to explain it.

## Materials and Methods

### The study area

Zamfara State of Nigeria is located between latitude 12°10'N and 12°167'N, and longitude 06 °15'E and 06°250'E, it has a total area of 39,762 square kilometers and a warm tropical climate with an average temperature rising to 38oC between March and May while the rainy season starts in late May to September with total annual rainfall ranging from 875 mm to 1,300 mm [24]. There are more than 21 artisanal gold mines in Zamfara state cutting across six Local Government Areas, covering about 219 KM2 [25] though some are no longer operating.

### Sample sites selection

**Figure 1.**
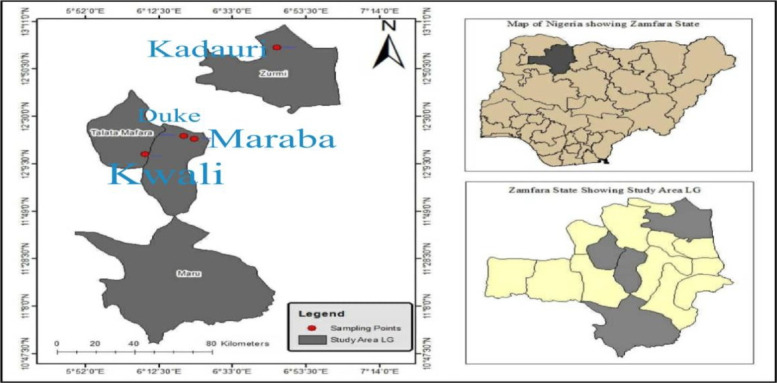
Map of Zamfara State, Nigeria showing the Sampling Locations.

Three gold mines namely Kwali (05°45.49'E–11°59.66'N), Duke (06°19.56'E–12°21.45'N) and Maraba (06°22.43'E–12°20.26'N) were randomly selected for this study according to [26] who through geostatistical and spatial analysis concluded that the optimal sampling scale should account for at least 3% of the total area of study. Kadauri (06°08.71'E–12°13.56'N), a non–mining and an upland agrarian area about 20 km away from the mining area, was also chosen as a control. Figure 1 shows the map of Zamfara state, Nigeria and the sampling locations during the study.

### Sample collection and analysis

Each gold mining site was divided into quadrants where sample locations were carefully chosen based on the size of the site after a pre–sampling survey, sampling spots were cleaned of debris while composite topsoil with depth from 0 to 15 centimeters were collected from different selected locations using soil auger so as to provide representative coverage of each mining site and the control area according to [27, 28]. There were 12 sampling spots in Kwali, 8 in Maraba, 7 in Duke and 8 in Kadauri from where the collected soil samples were homogenized and transferred into clean polythene bags which were immediately transported to the laboratory for heavy metals analyses, this was done monthly for a period of 12 months.

Soil samples were air dried for 48 hours, crushed into powder using sterile mortar and pestle, and then sieved through a 2mm mesh after which 1g was weighed into a 250ml glass beaker and 20ml nitric acid, hydrofluoric acid, and perchloric acid mixture in a ratio 3:1:1 was added to it. The mixture was then diluted to 50ml with distilled deionized water, heated on a hot plate at 95℃ for 1 hour and left to cool to room temperature, then filtered through Whatman No. 42 filter paper and the final volume made up to 50ml by adding distilled deionized water according to [29, 30]. The analysis for heavy metals: Pb, Cd, As, Ni, Cu, Cr, Mn, Zn, Co, Al, Fe and Au was done using atomic absorption spectrophotometer (AAS) (Shimadzu, model AA-6800, Japan) where the instrument was fitted with specific lamp for a particular metal and calibrated using blank and manually prepared standard solutions from a 1000 ppm stock solutions of each metal. Standard working conditions of the instrument including wavelength, slit width, lamp current and detection limits among others, were set for specific metals as specified by the manufacturers and presented in Table 1, digested samples were introduced into the spectrophotometer and their absorbance (A) were measured at their respective wavelengths against the blank solution (ΔA = As – Ab). This difference was used as an analytical signal to plot a calibration curve against concentrations in a series of standard working solutions from where the results were read in mg/kg with 3 replications to ensure accuracy according to [31].

### Quality control

All utensils used in the analyses of samples were washed with liquid soap and clean water, rinsed with distilled water and soaked overnight in 10 % nitric acid according to [32] and high pure (Anal R grade) chemicals and double distilled water were used for preparing solutions for analyses while relevant calibration solution (Analytic Jena) was used for each metal. All metal concentrations were measured in triplicates then average was taken and readings were recorded carefully to avoid parallax errors.

### Statistical analysis

One-way analysis of variance (ANOVA) was used to determine whether significant differences exist among the values of different heavy metals across the three mining areas and the control site. Pearson’s correlation analysis was conducted to measure the strength of the relationships between heavy metals within the samples while multivariate statistical techniques which include principal component analysis (PCA) was performed using DATAtab statistics software to assess the dynamics of metal distribution and identify the potential contamination sources.

### Heavy metals pollution indices and ecological risk Aassessment

Contamination factor (Cf) assesses soil contamination by heavy metals against the pre–anthropogenic activities reference levels as given by [33]. Cf of the analysed metals from different sampling locations were computed using the Hakanson formula:


(1) $$\mathrm{Cf}=\frac{\mathrm{Cn}}{\mathrm{Bn}}$$

Where: *C_n_* = metal concentration in the soil and Bn = background concentration given by Hakanson. The following gradations are given to evaluate the contamination factor: Cf < 1, no/low contamination; 1 ≤ Cf < 3, moderate; 3 ≤ Cf < 6, considerable; 6 ≤ Cf – very high contamination.

Geoaccumulation index (Igeo) is a quantitative measure of the extent of metal pollution in the soil and is calculated by computing the base 2 logarithm of the measured total concentration of the metal over its background concentration using the following equation postulated by [34]:


(2) $$I\mathrm{geo}=\log_{2}\,\frac{C_{n}}{1.5\times B_{n}}$$

Where *C_n_* is the measured concentration of the element in the samples, and Bn is the background or pristine value of the element as shown in Table 1, 1.5 = constant used to address variations of background data due to lithological changes. Igeo< 0 = practically unpolluted, 0 <Igeo< 1 = unpolluted to moderately polluted, 1 <Igeo< 2 = moderately polluted, 2 <Igeo< 3 = moderately to strongly polluted, 3 <Igeo< 4 = strongly polluted, 4 <Igeo< 5 = strongly to extremely polluted and Igeo>5 = extremely polluted.

Pollution load index (PLI) measures the severity of pollution and its variation along sites. It is a quick tool for comparing the pollution status of different places and gives the overall toxicity grade of every sampling site [30], it is calculated as:


(3) $$\sqrt[{n}]{\mathrm{Cf1}\,\times \mathrm{Cf2}\,\times \mathrm{Cf3}\ldots \times \mathrm{Cfn}}$$

Where: *Cf* is the contamination factor of metals and n is the number of metals assessed. The PLI value of >1 is polluted, whereas <1 indicates no pollution.

Potential ecological risk index (PERI) was calculated using the formula introduced by [35]:


(4) $$\mathrm{RI}=\sum_{i=1}^{n}E_{r}^{i}=\sum_{i=1}^{n}T_{r}^{i}\times \left(\frac{C}{C_{n}^{i}}\right)$$

Where: RI is the sum of the potential ecological risks of various heavy metals, $E_{r}^{i}$ is the RI of an individual element, C is the concentration of metals in the sample, $C_{n}^{i}$ is the background value of the elements in the soil which is considered from world average value in shale (mg kg–1) and the values are Fe = 47200, Zn = 95, Pb = 20, Co = 19, Cu = 45, Cr = 90, Ni = 68, Mn = 850, As = 13 and Cd = 0.3 (Edori and Kpee, 2017), $T_{r}^{i}$ is the toxic response factor of metals developed by Håkanson which are Cd = 30, As = 10, Pb = Cu = Ni = Co = 5, Cr = 2, Mn = Zn = Fe = 1 [36]. PERI < 40 = low risk, PERI 40–80 = moderate risk, PERI 80–160 = considerable, PERI 160–320 = high risk and PERI > 320 = significantly high risk.

## Results and Discussion

### Mean heavy metals concentration in soil from the study area

In Maraba, the mean concentration of Pb in the soil throughout the study period was 110.32 mg/kg with a standard deviation of 71.73 and a range of 16.09 – 201.94. Fe had the highest mean concentration of 117.62 ± 62.09 mg/kg, while Ni had the lowest mean concentration of 4.28 ± 3.74 mg/kg. The Metals occurred in the following hierarchy: Fe > Pb > Au > Al > Zn > Cu > Cd > Co > As > Mn > Cr > Ni. The mean concentration of Pb in soil from Duke was 130.62 ± 61.12 mg/kg with a standard deviation of 61.12. Pb concentration was the highest in Duke, while As had the lowest concentration of 1.94 ± 1.33 mg/kg. They occurred in the following order: Pb > Fe > Au > Al > Zn > Cd > Co > Cu > Ni > Mn > Cr > As. Kwali mining site had the mean Pb concentration of 148.59 ± 63.52 mg/kg in the soil which was the highest of all elements. In this mining area Co had the lowest concentration of 2.44 ± 2.83 mg/kg and the elements occurred in the order: Pb > Fe > Au > Al > Zn > Cd > Cu >Ni > As > Mn > Cr > Co. Kadauri the control site had the mean Pb concentration of 28.16 ± 17.62 mg/kg. The element with the highest mean concentration was Fe (37.45 ± 27.53 mg/kg) and the lowest was Ni with the mean concentration of 1.25 ± 1.30 mg/kg as they occurred in the following sequence: Fe > Pb > Au > Al > Zn > Mn > Cu > Co > Cr > As > Cd >Ni.

All metals except for Mn and Zn, differ significantly (P<0.05) across sampling stations with Kadauri the control site having lower values compared to the mining areas which showed the impact of mining activities on metal concentration in the soil. The entire mean levels of heavy metals in soil across sampling stations in comparison with the World Health Organization (WHO) [37–39] standard are presented in Table 2.

The concentration of metals in Kadauri the non-mining area could be as a result of the place being a lowland which is often flooded and also used for agriculture as it is an arable land. The mean concentrations of different metals across the sampling stations were by far above the (WHO) [37–39] standard for heavy metals in soil. This indicates a high level of contamination and poses an immediate and long term health risk for the miners as well as the villagers living in those communities as metals can be taken up by plants, water or air which will eventually end up in humans [40]. Metal concentrations above maximum permissible limits in soil around gold mining areas in Nigeria were also reported by [14] and [41] in their separate studies on mineral composition of mine tailings from those areas. These indicate a serious threat to the environment and the inhabitants if not properly checked and monitored.

The hierarchy of occurrence of metals in soil did not follow a stable trend but showed some similarities among the sampling stations which was dominated by Pb > Fe > Au > Al > Zn followed by other metals in a manner that is not fixed. This could be explained by their abundance as Fe and Al are part of the ten major elements that constitute over 99% of the total element content of the earth’s crust according to [42] and the mining area being a sedimentary rock, acts as an adsorbent for Pb and other metals which further catalyzes their accumulation through the precipitation of metal sulphides as explained by [43] in their review on heavy metals in contaminated soils. In almost all cases Pb and Fe occurred in higher concentrations than Au for which the mining activities take place [44]. in their studies on identifying pathfinder elements for gold in Wa–Laura belt, Northwest Ghana, also found out that gold concentration was lower than that of its associated elements and they inferred from the result that the occurrence of these elements can be linked to both primary dispersion from underlying rocks and secondary processes such as lateralization while the data showed strong associations between metals: Fe, Mn, Pb, Ag etc and suggested that these elements can be used as pathfinders for gold. [45] also reported that earth tested in a mining village in Zamfara state, Nigeria had Pb levels above 50,000 parts per million (ppm) which by far exceeded the 400 ppm USEPA limit for areas around playgrounds.

### Relationship between heavy metals in soil from the study area

**Figure 2.**
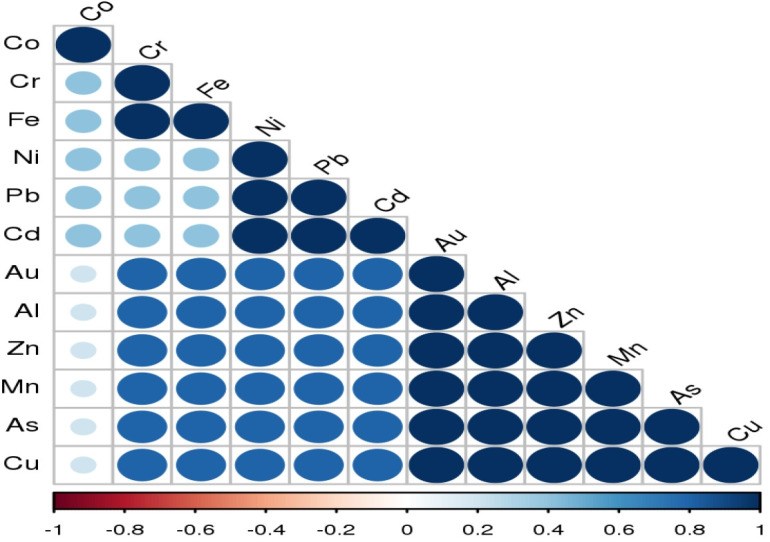
A correlogram showing the relationship between heavy metal pairs during the study: 0ositive correlations are displayed in blue and negative correlations in red color. Color intensity and the size of the circle are proportional to the correlation coefficients.

Pearson’s correlation analysis interestingly showed no negative relationship between all analysed heavy metal pairs but rather, there were strong positive relationships at 95 % level of confidence with r ≥ 0.5 in about 70 % of all metals, 28 % of which have r = 1 including Cr/Fe (P < 0.0001), Pb/Ni (P < 0.0001), Cd/Ni (P < 0.0001), Al/Au (P < 0.0001), Zn/Au (P < 0.0001), Mn/Au (P < 0.0001), As/Au (P < 0.0001) and others. Metal pairs with pairs with no significant positive correlation include Cr/Co (r = 0.42; P = 0.17), Fe/Co (r = 0.34; P = 0.28) and Cd/Fe (r = 0.43; P = 0.13) while the pairs Au/Co, Al/Co and Cu/Co had r = 0.00 with P = 1. The relationship between metal pairs during this study is presented in a correlogram in Figure 2. The high correlation coefficients in metal pairs indicate that they reach the soil at this location through a similar method [46] hence the concentration of one increase with the concentration of the other.

### Heavy metals pollution indices and ecological risk

Gold had the highest contamination factor in all mining areas and the non–mining area with the highest Cf being 20273.33 in Au from Kwali while Fe had the lowest Cf of 0.0008 in Kadauri sampling location. Pb, Cd, Al and Au had contamination factors > 1in all sampling locations with Pb in Kadauri having moderate contamination, Pb in Maraba and Cd in Kadauri having considerable contamination and Cd in Maraba, Duke and Kwali as well as Al and Au in all sampling locations having very high contamination. This implies that human activities in the area have raised the levels of these heavy metals above the pre–anthropogenic activities reference levels. The high Cf observed in gold may not be unconnected to the fact that the place is a gold mining area and it is the target element which all miners work to concentrate it through various processes, leading to its discharged in the soil according to [47]. This is similar to the findings of [48] who assessed the ecological risk of heavy metals in contaminated soils from selected villages in Zamfara state, Nigeria and found out that all the gold processing sites were moderate to severely contaminated with Pb and Fe.

The highest and lowest Igeo were 15.48 and 1.57 in Au and Cd respectively, both from Kadauri. Only Pb in Maraba, Duke and Kwali, Cd in Maraba, Duke and Kadauri together with Al and Au in all sampling locations had positive Igeo indicating accumulation above background values of these metals which resulted into moderate to extremely polluted environment. Heavy metals geoaccumulation indices during this study are presented in Table 3. [49] in his assessment of trace metal pollution indicators in soils around oil well clusters reported geoaccumulation index predominantly reflecting non-contamination for the different trace elements and sampling locations as Igeo values < 0 indicates a practically unpolluted environment. Positive Igeo values were reported in about 60% of the studied metals by [50] in Itakpe iron-ore mining area of Nigeria where the status of individual metals ranged from moderate to extremely polluted. The use of Igeo with its somewhat differentnature of calculation which involves logarithmic function and a background correction factor of 1.5 is more consistent and preferable [51].

Pollution load index (PLI) was highest (0.67) in Maraba and lowest (0.26) in Kadauri indicating no or low level of pollution in the area The PLI of heavy metals from different sampling locations during this study are shown in table 3. PLI is a complex index whose calculation requires total concentrations of all analyzed heavy metals as well as individual values of contamination factor to give a comprehensive degree of heavy metal pollution in an area [52]. [53] observed PLI less than 1 for all stations in their study of sediment quality of Benin River, Nigeria indicating practically uncontaminated condition while [54] reported PLI values above unity for all sites around landfills in Bulawayo, Zimbabwe.

### Heavy metals source apportionment

**Figure 3.**
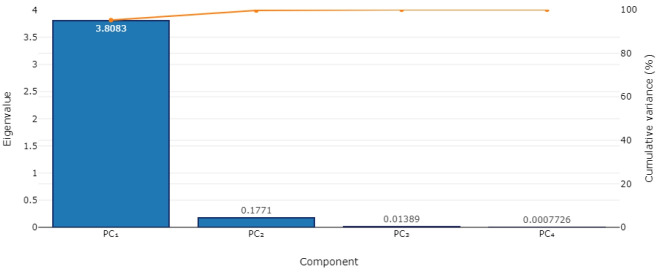
Principal components and their eigenvalues in Zamfara, Nigeria during the study.

**Figure 4.**
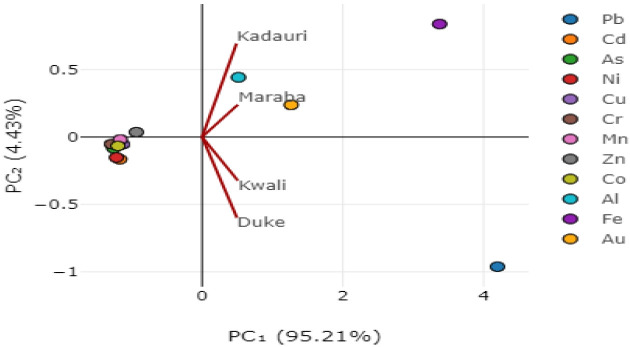
The two major principal components and their respective variance contributing elements in Zamfara, Nigeria during the study

Four principal components (PCs) accounted for the overall variations in heavy metals concentration in the study area with PC1 accounting for about 95.21 %, PC2 for 4.43 %, PC3 for 0.35 % and PC4 for about 0.02 %. PCs 1 and 2 constituted about 99.64 % of the total variation with the elements; Pb, Al, Fe and Au accounting for these variations in PC1 indicating the anthropogenic source of contaminants while Zn, Al, Fe and Au accounted for variations in PC2 suggesting the dominance of both natural and artificial contaminant sources. These results are similar to the findings of [59] who worked on heavy metals pollution levels in gold mining areas of Tanzania where it was found out that Pb, Cr, Cu, As, Mn and Ni were strongly associated with PC1 that originated from mining and related activities indicating mining as the source of contamination. The principal components together with their aegenvalues and the cumulative proportion of all values are presented in Table 4 while the component loadings and respective element contributions are presented in Figures 3 and 4. PC1 is related to the geological origin and natural processes such as pedogenesis, mineralization and soil hydrology which are further aggravated by anthropogenic activities with its high variability as heavy metal concentration increases with such activities like mining and agricultural practices that do take place in the study area [60]. The parent rocks are very old Pre–cambrian igneous and metamorphic rocks which birthed granite and metasediments laden with Pb, Mn, Fe, Au, Al, Cu, Cr and Cd among other elements that weather into soils endowed with valuable ores and minerals including gold making mining the most lucrative business and studies showed that the soils were greatly perturbed [61]. [62] studied the effect of weathering and mineralogy on the distribution of major and trace elements in Iran and found out that these elements were related primarily to the parent material type and concluded that the chemical composition of a soil are generally controlled by the lithology of its parent material. PC2 was considered as anthropogenic activities influenced factor as many studies reported these metals to be associated with land use patterns which include traffic, fertilizer application, smelting etc which are generally carried out in the study area [63–65]. Principal component analysis is a practice of multidimensional scaling that reduce the number of variables to several groups of individuals and explains the variance in the data by examining multivariate relationships of the principal component scores as proposed by Hotelling in 1933 [66]. It has some similarities with correlation or regression analyses where it detects the structure in the relationships of different variables that have been reduced to a considerable number [67]. Dimensionality is reduced in a dataset thereby creating linear combinations of the original variables hence non–linear relationships between variables cannot be identified by PCA [68]. The principal components (PCs) are orthogonal to one another i.e. correlation is exactly or close to zero for all pairs of PCs [69].

## Conclusions

The highest metal concentration during this study was Pb (148.59 ± 63.52 mg/kg) in Kwali mining area and the lowest was Ni (1.25 ± 1.30 mg/kg) in Kadauri the control area with the general hierarchy of occurrence being dominated by Pb > Fe > Au > Al > Zn followed by other metals in an unspecified manner. All metals differed significantly (P<0.05) across sampling stations except for Mn and Zn with Kadauri the control site having lower values and all the mean concentrations of different metals were by far above the World Health Organization (WHO) standard for heavy metals in soil which portrays a health risk for the inhabitants of the area. Pearson’s correlation analysis carried out between metal pairs showed strong positive relationship with r ≥ 0.5 in about 70% of these pairs indicating commonality of source and similar contamination method. Pb, Cd, Al and Au had contamination factors > 1in all sampling locations which implies that human activities in the area have raised the levels of these heavy metals above the pre–anthropogenic activities reference levels. Only Pb in Maraba, Duke and Kwali, Cd in Maraba, Duke and Kadauri together with Al and Au in all sampling locations had positive Igeo indicating accumulation above background values of these metals. The highest PERI was 782.79 in Kwali mining area and the lowest was 142.15 in Kadauri with the three mining areas having significantly high ecological risks while Kadauri had a considerable ecological risk. Four principal components (PCs) account for the overall variation in heavy metals concentration in this study with PC1 accounting for about 95.21% and PC2 for 4.43% making about 99.64% of the total variance while respective element variance contributions suggested that PC1 is associated with anthropogenic activities with Pb, Al, Fe and Au contributing to it and PC2 is a natural component due to lithogenic factors with Zn, Al, Fe and Au being responsible for its variations. The study further brought to light the influence of artisanal gold mining on heavy metals concentration in soil which need to be regulated and safety measures be put in place. It also form a basis for further studies as these metals can be absorbed in plants, animals and water bodies with little or nothing being done to curb such occurrences in the area. It is therefore strongly suggested that authorities at the local and international levels should canvass support for research in heavy metals management and remediation in soil, awareness and regulation for the miners as well as safety facilities to curtail emergency situations in the study area.

## Figures and Tables

**Table 1.** Standard Working Conditions of (Shimadzu, model AA-6800, Japan) Atomic Absorption Spectrophotometer.

Element	Wavelength (nm)	Detection limit (mg/L)	Lamp current (mA)	Slit width (nm)
Pb	283.3	0.04	10	0.5
Cd	228.8	0.006	8	0.5
As	193.7	0.001	12	1.0
Ni	232.0	0.1	4	0.2
Cu	324.8	0.01	4	0.5
Cr	357.9	0.01	10	0.5
Mn	285.2	0.02	8	0.5
Zn	213.9	0.008	8	0.5
Co	240.7	0.010	7	0.2
Al	324.8	0.0015	11	1
Fe	248.3	0.065	5	0.2
Au	242.8	0.005	4	0.2

**Table 2.** Mean metal concentrations (mg/kg) in soil from gold mining areas of Zamfara State, Nigeria, their ANOVA test and comparison with WHO standards.

Metals	Maraba	Duke	Kwali	Kadauri	P–test	Inference	WHO limit	Reference
Pb	110.32 ± 71.73	130.62 ± 61.12	148.59 ± 63.52	28.16 ± 17.62	P < 0.05	Diff. sig.	0.01	[36]
Cd	5.67 ± 2.19	6.81 ± 3.21	7.39 ± 3.05	1.33 ± 1.17	P < 0.05	Diff. sig.	0.003	[37]
As	4.95 ± 4.24	1.94 ± 1.33	6.03 ± 3.42	1.37 ± 0.68	P < 0.05	Diff. sig.	0.01	[36]
Ni	4.28 ± 3.74	5.61 ± 3.22	6.36 ± 4.86	1.25 ± 1.10	P < 0.05	Diff. sig.	0.02	[36]
Cu	5.72 ± 2.69	5.67 ± 4.39	6.56 ± 4.31	2.92 ± 1.96	P < 0.05	Diff. sig.	1.00	[36]
Cr	4.37 ± 2.04	2.08 ± 1.99	2.71 ± 1.91	1.61 ± 0.99	P < 0.05	Diff. sig.	2.00	[36]
Mn	4.94 ± 3.37	4.31 ± 2.79	5.47 ± 4.17	3.13 ± 2.27	P > 0.05	Not sig.	0.05	[37]
Zn	8.67 ± 5.32	8.36 ± 5.06	9.63 ± 4.28	5.40 ± 3.17	P > 0.05	Not sig.	3.00	[36]
Co	5.53 ± 2.98	6.11 ± 3.64	2.44 ± 1.83	2.34 ± 1.62	P < 0.05	Diff. sig.	5.00	[38]
Al	36.31 ± 19.23	30.76 ± 14.63	36.59 ± 16.01	19.58 ± 11.73	P < 0.05	Diff. sig.		
Fe	117.62 ± 62.09	69.79 ± 22.79	102.01 ± 39.41	37.45 ± 17.53	P < 0.05	Diff. Sig.	0.30	[38]
Au	58.78 ± 15.48	45.14 ± 11.82	60.82 ± 14.11	20.57 ± 5.12	P < 0.05	Diff. Sig.		

The highest PERI was 782.79 in Kwali mining area and the lowest was 142.15 in Kadauri with the three mining areas having significantly high ecological risks while Kadauri had a considerable ecological risk. Potential ecological risk indices of heavy metals from the sampling locations during this study are resented in Table 3. Potential ecological risk index provides a quantitative approach to the extent of potential hazards in an area based on the comprehensive considerations of ecological and environmental effects and individual heavy metals toxicity [55]. [56] reported low ecological risks in paddy fields of Fujian province, China while [57] reported significantly high ecological risk index in a mining area in China. High ecological risk indices of heavy metals in an area means the inhabitants are constantly exposed to threats which have the potential to cause harm [58].

**Table 3.** Heavy metals pollution indices in soil from gold mining sites of Zamfara State, Nigeria.

HeavyMetal	Maraba		Duke		Kwali		Kadauri	
	Cf	Igeo	Cf	Igeo	Cf	Igeo	Cf	Igeo
Pb	5.52	1.92	6.53	2.12	7.43	2.31	1.41	- 0.09
Cd	18.90	3.66	22.70	3.92	24.63	- 2.64	4.43	1.57
As	0.38	- 2.0	0.15	- 3.47	0.46	- 1.69	0.11	- 3.84
Ni	0.06	- 4.64	0.08	- 4.06	0.09	- 4.06	0.02	- 9.97
Cu	0.13	- 3.47	0.13	- 3.64	0.15	- 3.47	0.07	- 4.64
Cr	0.05	- 5.06	0.02	- 5.64	0.03	- 5.64	0.02	- 6.64
Mn	0.006	- 7.97	0.005	- 8.38	0.006	- 7.97	0.004	- 8.38
Zn	0.09	- 4.06	0.09	- 4.06	0.10	- 3.84	0.06	- 4.64
Co	0.29	- 2.39	0.32	- 2.25	0.13	- 3.47	0.12	- 3.64
Al	55.86	5.22	47.32	4.98	56.29	5.23	30.12	4.33
Fe	0.003	- 8.97	0.002	- 10.12	0.002	- 9.97	0.0008	- 10.97
Au	19593.33	13.67	15046.67	13.29	20273.33	13.72	6856.67	15.48
PLI	0.67		0.54		0.65		0.26	
PERI	620.19		717.94		782.79		142.15	

**Table 4.** Principal components and total variance for heavy metals in gold mining areas of Zamfara State, Nigeria.

PC summary	PC1	PC2	PC3	PC4
Eigenvalues	3.8083	0.1771	0.01389	0.0007726
Proportion of variance	95.21 %	4.43 %	0.35 %	0.02 %
Cumulative proportion of variance	95.21 %	99.64 %	99.98 %	100 %
